# Human Liver Organoids as an Experimental Tool to Investigate Lipocalin-2 in Hepatic Inflammation

**DOI:** 10.3390/cells15030216

**Published:** 2026-01-23

**Authors:** Katharina S. Hardt, Robert F. Pohlberger, Diandra T. Keller, Eva M. Buhl, Florian W. R. Vondran, Anjali A. Roeth, Ralf Weiskirchen, Sarah K. Schröder-Lange

**Affiliations:** 1 Institute of Molecular Pathobiochemistry, Experimental Gene Therapy and Clinical Chemistry (IFMPEGKC), RWTH University Hospital Aachen, D-52074 Aachen, Germany; khardt@ukaachen.de (K.S.H.); dikeller@ukaachen.de (D.T.K.); 2 Clinic for General, Visceral, Pediatric and Transplant Surgery, RWTH University Hospital Aachen, D-52074 Aachen, Germany; rpohlberger@ukaachen.de (R.F.P.); fvondran@ukaachen.de (F.W.R.V.); aroeth@ukaachen.de (A.A.R.); 3 Electron Microscopy Facility, Institute of Pathology, RWTH University Hospital Aachen, D-52074 Aachen, Germany; ebuhl@ukaachen.de

**Keywords:** Lipocalin-2 (LCN2), patient-derived organoid, liver, inflammation, in vitro model, liver disease

## Abstract

**Highlights:**

**What are the main findings?**

- Patient-derived liver organoids successfully mimic key architectural features of hepatocytes and exhibit self-renewing potential, making them a valuable in vitro model for studying lipocalin-2 (LCN2) in hepatic inflammation.
- LCN2 expression is significantly upregulated in these organoids under inflammatory conditions, particularly through stimulation with interleukin-1β (IL-1β) and tumor necrosis factor alpha (TNF-α), indicating their responsiveness to pro-inflammatory signals.

**What are the implications of the main findings?**

- The establishment of human liver organoids as a model provides new opportunities for investigating the role of LCN2 in liver pathologies, potentially aiding in the development of patient-specific treatment strategies for diseases such as metabolic dysfunction-associated steatotic liver disease (MASLD) and hepatocellular carcinoma (HCC).
- Understanding the signaling pathways involved in LCN2 regulation may contribute to therapeutic advancements targeting inflammatory processes in liver diseases, enhancing personalized medicine approaches.

**Abstract:**

The 25 kDa glycoprotein lipocalin-2 (LCN2) is widely expressed and has diverse functions, ranging from physiological to pathophysiological processes. In the liver, LCN2 is primarily associated with inflammatory processes and is considered a potential biomarker in metabolic disorders. However, a significant challenge is the absence of a suitable human in vitro model for studying LCN2 and its associated signaling pathways. Therefore, we have successfully generated patient-derived liver organoids of both male and female origin, providing a novel in vitro model for LCN2 research. Our data show that the self-renewing organoids mimic essential architectural features of hepatocytes, as demonstrated by electron microscopy and F-actin staining. Consistent with the expression profile observed in liver tissue, the isolated 3D organoids exhibit minimal endogenous LCN2 levels. Next, the LCN2 expression was studied at the protein and mRNA levels under inflammatory conditions by treating the organoids with various cytokines and lipopolysaccharides (LPS). Our results show that LCN2 expression is significantly upregulated by IL-1β and TNF-α in an NF-κB-dependent manner, but remains unchanged with IL-6 or LPS. In conclusion, we have established human patient-derived liver organoids as a valuable model for investigating LCN2 signaling mechanisms. This study lays the foundation for future research on the role of LCN2 in liver pathologies, aiding in disease progression understanding and facilitating patient-specific treatment predictions.

## 1. Introduction

The lipocalin superfamily comprises various proteins whose primary function is the transport of hydrophobic molecules [1]. They share a conserved three-dimensional structure composed of a single β-barrel configuration with eight antiparallel strands. The barrel forms a central groove that binds ligands of different sizes and shapes [2]. Lipocalin-2 (LCN2), also known as neutrophil-gelatinase associated lipocalin (NGAL), or its murine orthologue 24p3 protein, is a prominent member of the lipocalin superfamily [3]. LCN2 is a glycoprotein with a molecular weight of 25 kDa, expressed in nearly all tissues [4]. It plays diverse roles in both physiological and pathological processes. During microbial infections, it sequesters iron-loaded siderophores, thereby restricting bacteria’s access to iron and inhibiting their proliferation [5]. Furthermore, LCN2 functions range from implications in cell apoptosis, associations with lipid metabolism, and cell migration [6]. In the liver, it is associated with inflammatory processes leading to an upregulation of *LCN2* mRNA and protein expression, with hepatocytes as the major source [4,7]. Based on various studies, LCN2 is also being discussed as a possible biomarker for liver diseases, ranging from metabolic dysfunction-associated steatotic liver disease (MASLD), metabolic dysfunction-associated steatohepatitis (MASH), to hepatocellular carcinoma (HCC) [6]. In particular, it is assumed that LCN2 has a protective function in steatohepatitis. Compared to a healthy liver, in which barely any *LCN2* mRNA or protein expression is detectable, LCN2 is significantly overexpressed in HCC tumors [8].

Most studies investigating the physiological and pathophysiological functions of LCN2 have used rodent models, particularly mice, so far. Not only for animal welfare reasons within the framework of the 3R regulation to reduce, replace, and refine animal testing [9], but also because human and murine LCN2 share only approximately 62% identical amino acid sequence, it is important to establish alternative models to understand the function of LCN2 in the human liver [10]. Established hepatocyte cell lines, such as HepG2 or HuH7, are particularly suitable for molecular biological processes of LCN2 expression [1,11]. However, these immortalized cell lines, which originate from liver tumors, exhibit altered gene expression and also functional alterations [12]. Alternatively, primary hepatocytes isolated from tissue circumvent the tumorigenicity of cell lines, but cannot be cultivated permanently due to their loss of proliferative capacity and therefore offer only limited possibilities [13]. Over the past few years, various 3D models have been developed to avoid the problems associated with conventional 2D cell culture. For this purpose, liver organoids were generated from patient-derived liver biopsies as a promising model system [14]. Organoids are defined as self-organizing, three-dimensional systems derived from (pluripotent) stem cells, progenitor, or differentiated cells, in which coordinated cell–cell and cell–matrix interactions reproduce essential aspects of native tissue organization and function under in vitro conditions [15]. Therefore, these 3D structures have been shown to better mimic the functions and architecture of in vivo tissues than conventional 2D cell culture.

As of now, the LCN2 expression and signaling have been investigated only in primary hepatocytes or tumor-derived cell lines. The study aimed to establish 3D organoids generated from liver biopsies as an alternative in vitro model. We showed for the first time that patient-derived organoids express *LCN2* mRNA and protein. Thereby, LCN2 expression can be induced under inflammatory conditions in an NF-κB-dependent manner. This finding classifies organoids as reliable tools for further studies on LCN2 and its role in the progression of physiological and pathological conditions.

## 2. Materials and Methods

### 2.1. Isolation and Passaging of Liver Organoids

Organoids were established from liver biopsies taken from male and female patients during surgical procedures at the University Hospital RWTH Aachen. Ethical standards were approved beforehand (EK206/09). The initiation of human liver organoids followed the protocol of Broutier et al. [14]. To isolate hepatocytes, liver tissue was digested with collagenase D (#11088858001, Roche Diagnostics, Rotkreuz, Switzerland) and DNase I (#10104159001, Roche Diagnostics), followed by washing with cold serum-free standard Dulbecco’s Modified Eagle Medium (DMEM High Glucose, D6171, Sigma-Aldrich, Merck, Taufkirchen, Germany). Single cells were embedded in Geltrex^TM^ (A1413202, Gibco, Thermo Fisher Scientific, Waltham, MA, USA) as an extracellular matrix and seeded into 24-well suspension culture plates (662102, Greiner Bio-One GmbH, Frickenhausen, Germany). For the first 7 days post-isolation, cells were cultured in organoid initiation medium (#100-0384, StemCell Technologies, Vancouver, BC, Canada) supplemented with 10 mM Rho kinase inhibitor (#ab120129, Abcam, Cambridge, UK). Subsequent cultivation was done in organoid growth medium (OGM, #100-0385 StemCell Technologies) with 1× penicillin-streptomycin (P0781, Sigma-Aldrich). To subculture organoids, the extracellular matrix was dissolved and washed off with cold serum-free standard DMEM (D6171, Sigma-Aldrich, Merck), supplemented with 4 mM L-glutamine (G7513, Sigma Aldrich, Merck) and 1× penicillin-streptomycin. Organoids were fragmented, reseeded in the extracellular matrix, and cultivated in OGM, with a passaging ratio of 1:4 weekly. The medium was changed once a week. For experiments, organoids from middle to late passages (passage 5 to 14) were used, with at least five different donors represented. For live cell imaging, freshly passaged organoids were incubated in the ImageXpress^®^ Pico automated cell imaging system (Molecular Devices, San Jose, CA, USA, Version 2.6.7354.30739) for 72 h.

### 2.2. Transmission Electron Microscopy

For transmission electron microscopy (TEM), organoids were fixed for at least one hour at room temperature (RT) using a phosphate-buffered saline (PBS) solution containing 3% glutaraldehyde and washed in 0.1 M Soerensen’s phosphate buffer (Sigma-Aldrich, Merck). The organoids were then post-fixed in 25 mM sucrose buffer (Sigma-Aldrich) containing 1% osmium tetroxide (Carl Roth, Karlsruhe, Germany). Subsequently, the organoids were dehydrated using an increasing alcohol series. The procedure included a 30 min incubation in propylene oxide (Serva, Heidelberg, Germany), followed by a 1 h incubation in a 1:1 mixture of Epon resin and propylene oxide, and an additional 1 h incubation in pure Epon. Epon polymerization was then carried out at 90 °C for 2 h. Samples were then cut into ultrathin slices (70–100 nm) using an ultra-microtome (Reichert Ultracut S, Leica, Wetzlar, Germany) with a diamond knife (Diatome Ltd., Nidau, Switzerland). These slices were picked up on Cu/Rh grids (HR23 Maxtaform, Plano GmbH, Wetzlar, Germany). To enhance contrast, the samples were stained with 0.5% uranyl acetate and 1% lead citrate (both from Science Services, Munich, Germany). The samples were viewed using a Zeiss Leo 906 (Carl Zeiss AG, Oberkochen, Germany) transmission electron microscope at an acceleration voltage of 60 kV.

### 2.3. Cytokine and Inhibitor Treatment

For experiments involving treatment with cytokines and lipopolysaccharides (LPS), the organoids were cultured for seven days post-passaging. Subsequently, the organoids were treated with the following final concentrations of the respective substances: 2.5 ng/mL IL-1β (130-093-895, Miltenyi Biotec, Bergisch Gladbach, Germany), 10 ng/mL TNF-α (210-TA, R&D Systems, Wiesbaden, Germany), 10 ng/mL IL-6 (130-093-929, Miltenyi Biotec), and LPS 10 µg/mL (L-6143, Sigma-Aldrich, Merck). All compounds were diluted in OGM. In the case of signaling pathway inhibition, the inhibitors QNZ (545380-34-5, Santa Cruz Biotechnology, Heidelberg, Germany), JNK-IN-8 (SML1246, Sigma-Aldrich, Merck), and SB203580 (559389, Calbiochem, Merck) were added 1.5 h before stimulation with IL-1β. Unless otherwise stated, organoids were harvested after 24h for RNA or protein isolation.

### 2.4. Western Blot Analysis

To analyze proteins from the whole organoid, the extracellular matrix was first dissolved in cold PBS and then removed by repeated washes. Subsequently, the organoids were lysed in RIPA buffer, with the addition of cOmplete^TM^ protease inhibitor cocktail (11849300, Roche) and phosphatase inhibitor cocktail 2 (P-5726, Sigma-Aldrich). The protein concentration of each sample was determined using a DC protein assay (5000116, Bio-Rad Laboratories, Feldkirchen, Germany). An equal amount of protein lysates (30 µg protein) was then mixed with Nu-PAGE^TM^ LDS sample buffer (NP0008, Invitrogen, Thermo Fisher Scientific) and dithiothreitol (DTT) as a reducing agent and finally boiled at 80 °C for 10 min to denature the proteins. Consequently, the same quantity of protein lysate was loaded onto a 4–12% gradient Bis-Tris gel (NP0322BOX, Invitrogen) and separated using a MES running buffer. Protein separation was followed by transfer to a 0.45 µm nitrocellulose membrane (GE10600001, Amersham, Cytvia, Marlborough, MA, USA) in NuPAGE blotting buffer. Non-specific binding sites were blocked by incubation with a 5% (*w*/*v*) non-fat milk solution in Tris-buffered saline (TBS) supplemented with 0.1% Tween-20 (M-TBST). The membranes were incubated overnight at 4 °C with primary antibodies (Table S1) diluted in 2.5% M-TBST or 2.5% BSA-TBST for phosphorylation-specific antibodies. Following washing and incubation with horseradish peroxidase-coupled secondary antibodies, the chemiluminescence signal was visualized using the SuperSignal West Dura extended duration substrate (34076, Thermo Fisher Scientific) and the iBright^TM^ FL500 imaging system (Invitrogen, Thermo Fisher Scientific).

### 2.5. RT-qPCR and RT-PCR-RNA Analysis

RNA was isolated using the PureLink^TM^ RNA Mini Kit (12183025, Invitrogen, Thermo Fisher Scientific) according to the manufacturer’s instructions, including additional DNase digestion (12185010, Invitrogen, Thermo Fisher Scientific). After RNA clean-up, 1 µg of RNA was reverse transcribed to complementary DNA (cDNA) using Superscript II reverse transcriptase (18064022, Invitrogen, Thermo Fisher Scientific) and random primers (C1181, Promega, Madison, WI, USA). For RT-qPCR, 25 ng of cDNA and 20 pmol of forward and reverse primers diluted in RNAse-free H_2_O were mixed with 20 µL SYBR-GreenER^TM^ qPCR Super Mix Universal (11760500, Thermo Fisher Scientific). Primer sequences are listed in Table S2. RT-qPCR cycle conditions were 10 min at 95 °C for initial denaturation and 40 amplification cycles with 15 s at 90 °C and 1 min at 60 °C. Each sample was measured in technical duplicates. Relative mRNA expression was calculated using the 2^−ΔΔCT^ method [16], normalized to *GAPDH* expression.

For RT-PCR, cDNA was amplified using recombinant Taq DNA polymerase (11146165001, Roche), diluted in 10× PCR buffer with added MgCl_2_ and dNTP mix (11277049001, Roche). Amplification was performed for 30 cycles with the following conditions: 95 °C for 5 min, 95 °C for 1 min, 60 °C for 1 min, 72 °C for 3 min, and 72 °C for 10 min. Afterwards, samples were separated on a 3% agarose gel supplemented with MIDORI Green Advance (MG04, Nippon Genetics Europe GmbH, Düren, Germany) for 1 h at 100 V and visualized with the iBright^TM^ FL500 imaging system (Invitrogen, Thermo Fisher Scientific).

### 2.6. Next-Generation Sequencing

For Next-Generation Sequencing (NGS) analysis of organoids, RNA was isolated as described in Section 2.5. The RNA yield and integrity were measured using UV spectrophotometry and an Agilent 4200 TapeStation. For reverse-transcription of rRNA-depleted samples, NEBNext Multiplex Oligos for Illumina (Index Primers Set1) were used. All library preparation, sequencing and primary analysis were performed at the IZKF Genomics Facility at the University Hospital RWTH Aachen. Libraries were sequenced on an Illumina MiSeq using v2 300-cycle (MS-102-2002, Illumina, SanDiego, CA, USA) chemistry.

### 2.7. Immunofluorescence Staining

For whole-mount immunofluorescence staining, organoids were seeded in 50 µL of Geltrex on 4-well culture slides (354114 Corning Inc., Falcon, Corning, NY, USA). The organoids were fixed with 3.7% paraformaldehyde for 1 h in the dark at room temperature and then permeabilized with a 0.1% Triton-X 100 solution at 4 °C overnight. Non-specific binding sites were blocked with 3% donkey serum in PBS for 30 min at room temperature. The primary antibody against LCN2 (AF1757, R&D Systems) was diluted 1:100 in blocking buffer and incubated overnight at 4 °C. Goat IgG (AB-108-C, R&D Systems) was used as a control at the same concentration. The following day, organoids were incubated with donkey anti-goat IgG (H+L), highly cross adsorbed secondary antibody Alexa Fluor 488 (A32814, Invitrogen, Thermo Fisher Scientific) at a 1:500 dilution in blocking buffer for 1 h at room temperature in the dark. For phalloidin staining, organoids were incubated with either Phalloidin-Rhodamine or Phalloidin-Alexa Fluor 488 (#R415 and A12379 Invitrogen, Thermo Fisher Scientific) for 30 min at room temperature in the dark, immediately after blocking non-specific binding sites. Nuclei were counterstained with 200 ng/mL of 4′,6-diamidino-2-phenylindole dihydrochloride (DAPI, #D1306, Thermo Fisher Scientific) for 10 min at room temperature. Finally, coverslips were mounted with Permafluor Aqueous Mounting Medium (TA-006-FM, Thermo Fisher Scientific). Images were acquired with a Nikon Eclipse E80i fluorescence microscope (Nikon, Düsseldorf, Germany) equipped with the NIS-elements Vis software (Software version 3.22.01).

### 2.8. Hematoxylin and Eosin Staining and Immunohistochemistry

Hematoxylin and eosin (HE) staining and immunohistochemistry (IHC) were performed according to established protocols [17]. In brief, for HE staining agarose-embedded organoids were formalin-fixed and paraffin-embedded. Slices were dehydrated in a declining alcohol series, stained with Mayer’s hematoxylin (Lillie’s Modification, S3309, DAKO, Santa Clara, CA, USA) and Eosin Y solution (HT1102116, Sigma-Aldrich) at a pH of 4.5, rehydrated and mounted with DPX mounting medium (06522, Sigma-Aldrich). For IHC slices, dehydration was followed by antigen retrieval with citrate buffer and blocking of avidin-biotin binding sites (X0590, DAKO). Unspecific binding sites were blocked with 5% rabbit serum (Y0902, DAKO) in a solution of 1% BSA, 0.1% cold water fish gelatin, 0.1% Triton-X-100 and 0.05% Tween-20. The primary antibody against LCN2 (1:40, AF1575, R&D Systems) diluted in blocking buffer was incubated overnight at 4 °C in a dark chamber. Endogenous peroxidase was blocked with a 3% H_2_O_2_ solution before adding the secondary antibody (1:300, E0466, DAKO). Expression of LCN2 was visualized through a DAB reaction (D9292, Sigmafast, Sigma-Aldrich) and counterstained using a 0.1% Seed red solution (10264, Morphisto, Offenbach am Main, Germany). Slides were mounted with DPX mounting medium, and images were taken with a Nikon Eclipse E80i microscope.

### 2.9. Statistical Analysis

Statistical analysis was conducted using GraphPad Prism 8 (GraphPad Software 8.4.2, Boston, MA, USA). The Shapiro–Wilk test was used to assess the normal distribution of values. If a Gaussian distribution was assumed, an ordinary one-way ANOVA was conducted without pairing of samples and assuming equal standard deviation (SD) among samples. The Tukey test was selected as the post hoc test for correcting multiple comparisons. If a Gaussian distribution was not assumed, a non-parametric Kruskal–Wallis test was performed with Dunn’s post hoc test correction for multiple comparisons. Data is presented as mean ± SD. Statistically significant results are denoted with an asterisk: * *p* < 0.05, ** *p* < 0.01, *** *p* < 0.001, **** *p* < 0.0001.

## 3. Results

### 3.1. Self-Renewing Human Liver Organoids

**Figure 1:**
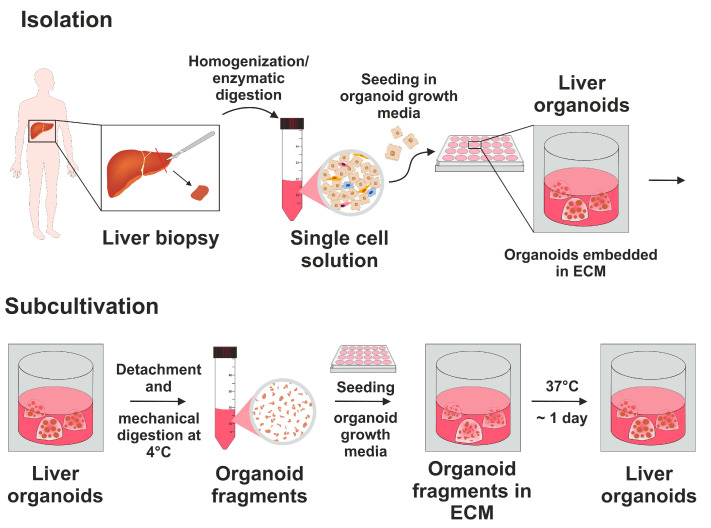
Isolation and subcultivation of patient-derived liver organoids. Isolation and subcultivation of organoids was conducted following the method outlined by Broutier et al. [14]. During the isolation process, liver biopsies were obtained during a surgical procedure. The tissues were then digested into single cells through homogenization and enzymatic digestion. These single cells were then embedded into an extracellular matrix (ECM) and cultivated in organoid initiation medium. Subsequently, for the subcultivation of the organoids, ECM was detached, and the organoids were mechanically divided into fragments. These fragments were then re-embedded into the ECM and cultured in organoid growth media.

As an alternative to conventional hepatocyte cell lines or primary human hepatocytes, organoids, which are 3D structures derived from patient biopsies were successfully generated. The patients consisted of 42.10% females and 57.90% males with an average age of 66.17 ± 7.35 years. A schematic overview of the isolation process and subculture of established organoids is shown in Figure 1. Organoid cultures that were established were used for experiments from passage 5 to 14.

**Figure 2:**
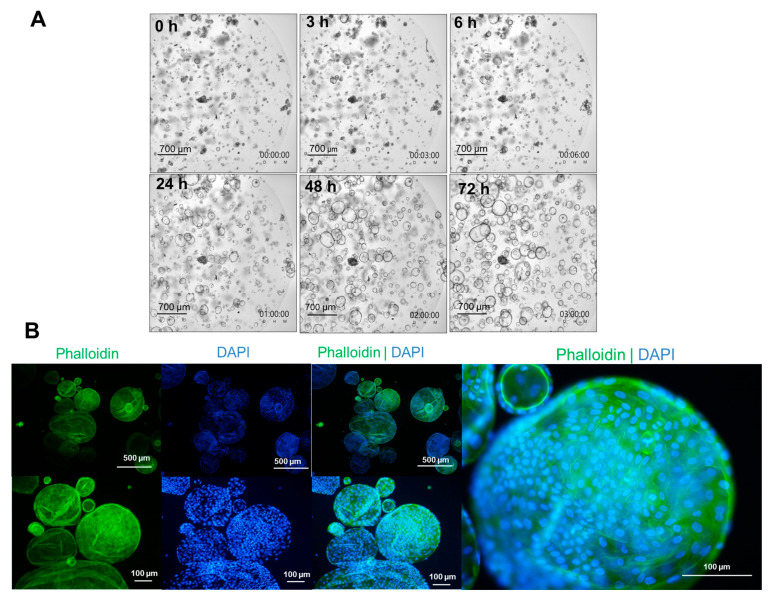
Microscopic imaging of human liver organoids. (**A**) Bright-field images were captured using an automated cell imaging system of freshly passaged organoids at various time points: 3 h, 6 h, 24 h, 48 h, and 72 h after cultivation. Organoid fragments begin to form new organoids between 6 and 24 h. The scale bars are equivalent to 700 µm. (**B**) Phalloidin staining (green) was utilized to display F-actin cytoskeletal structures in human liver organoids. Cell nuclei were stained with DAPI (blue). Images were taken using a Nikon Eclipse 80i with magnifications of 40×, 100× and 200×. F-actin structures are clearly visible between individual cells within a fully formed organoid. The scale bars are 100 µm or 500 µm.

For continuous cultivation and various experiments, it was necessary to subculture the organoids. The formation and growth of the organoids were continuously monitored over 72 h using a live-cell imaging system (Video S1). During this process, the organoids were fragmented, but gradually reassembled over time (Figure 2A). After 24 h, nearly all fragments built new organoids, which increased in size and reached a diameter of up to 500 µm after 72 h of cultivation. Our data clearly demonstrate their suitability as a self-renewing hepatocellular in vitro model.

The structural organization (F-actin filaments) of the organoids was visualized using a Phalloidin Alexa Fluor 488 probe (Figure 2B). Microscopic images of the organoids obtained via live-cell imaging and phalloidin staining showed the organoids’ varying diameters, ranging from approximately 50 µm to 500 µm. Phalloidin staining highlighted the cell–cell contacts of the individual cells in the overall 3D structure of the organoids. However, no individual structural filaments were visible within the cells. Together, bright-field imaging and F-actin staining gave a first impression of the architectural organization of the organoids.

### 3.2. Organoids Reflect Hepatocyte Ultrastructure in the Liver

**Figure 3:**
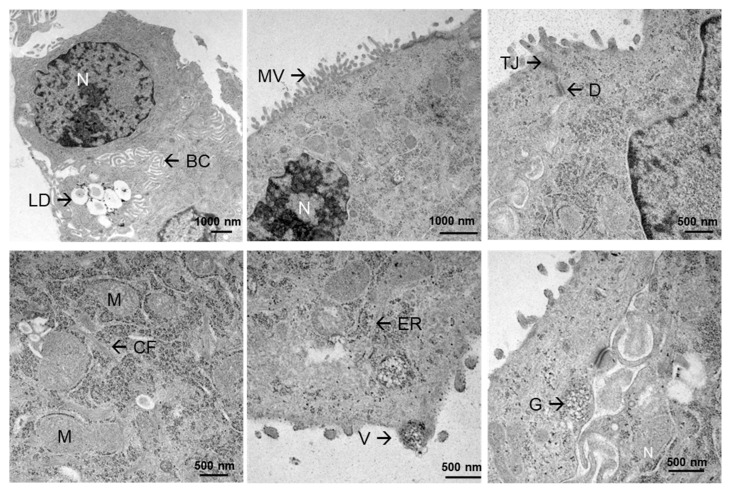
Electron microscopy showing the ultrastructure of human liver organoids. The organoids exhibit typical structures of hepatocytes found in the liver, such as lipid droplets (LD), bile canaliculi (BC), microvilli (MV), tight junctions (TJ), desmosomes (D), cytoskeletal filaments (CF), vesicles (V), glycogen (G) as well as typical cellular organelles like the nucleus (N), mitochondria (M) and endoplasmic reticulum (ER). The images were captured at magnifications of 7750×, 12,930×, 10,000×, 21,560×, and 27,800×. The scale bar indicates either 1000 nm or 500 nm.

To gain a more detailed view of the ultrastructure of the established organoids, TEM was performed. TEM clearly showed that the organoids exhibited the typical structure of hepatocytes in the liver (Figure 3). In addition to common cell organelles such as mitochondria (M), a central cell nucleus (N), and an endoplasmic reticulum (ER) with many ribosomes were visible. Specific hepatocellular structures were also observed, including the formation of lipid droplets (LD), microvilli (MV), cytoskeletal filaments (CF), and glycogen (G). Additionally, cell–cell contacts such as tight junctions (TJ) and desmosomes (D) were present between adjacent cells. Functional features included the formation of vesicles (V) and their secretion. The hepatocyte structure was particularly pronounced due to the formation of bile canaliculi (BC). Overall, the presence of these organelles reflected a typical liver-like appearance of hepatocytes.

### 3.3. Induction of LCN2 Expression Under Inflammatory Conditions

**Figure 4:**
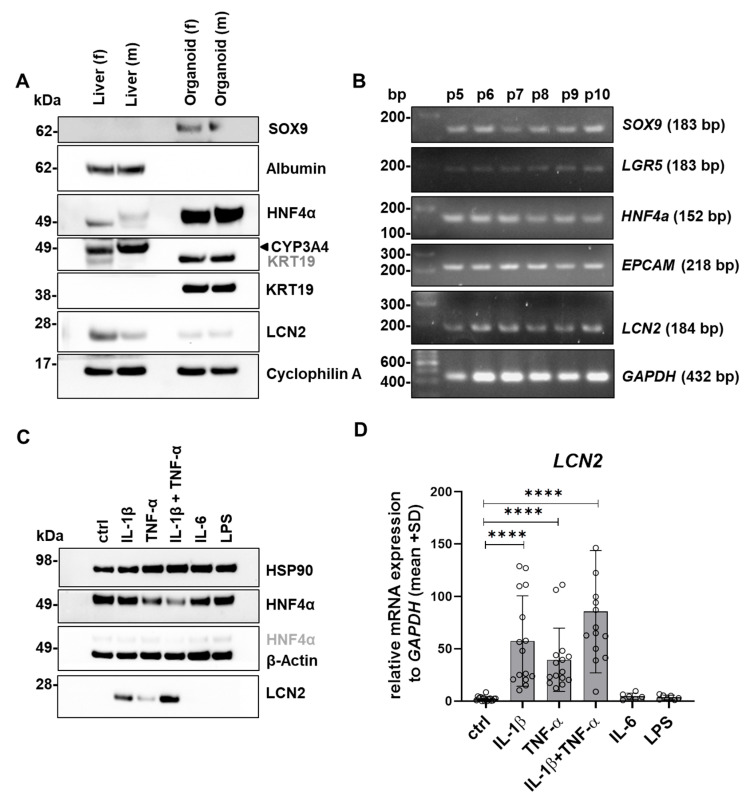
LCN2 expression in human liver organoids. (**A**) Comparison of protein expression of various liver-specific proteins between male (m) and female (f) liver tissue and organoid lysates. For liver tissue, 80 µg of protein lysate was used, while for organoids, 40 µg of protein lysates were applied for Western blotting. (**B**) mRNA expression of exemplary genes over a cultivation period of 6 passages is shown. All genes are stably expressed during long-term cultivation of organoids. (**C**) LCN2 protein expression and (**D**) mRNA expression of *LCN2* increase after stimulation of organoids for 24 h with certain inflammatory cytokines. The concentrations of cytokines used are 2.5 ng/mL IL-1β, 10 ng/mL TNF-α, 10 ng/mL IL-6, and 10 µg/mL LPS. HSP90, Cyclophilin A and β-Actin protein expression in (**A**,**C**) are used as controls. Data are shown as mean ± SD (n ≥ 6). Please note that signals labeled in grey resulted from previous probing. Multiple comparisons of data were performed to unstimulated (ctrl) samples. Statistical significances are highlighted with asterisks, **** *p* < 0.0001.

In order to use human liver organoids as an in vitro model, protein expression in female and male livers was compared with that in the respective organoids. As shown in Figure 4A, the organoids strongly express the hepatocyte nuclear factor 4 alpha (HNF4α), which is also a common marker of hepatocytes in the liver [18]. Additionally, the progenitor marker SRY-box transcription factor 9 (SOX9) [19] as well as the cholangiocyte marker cytokeratin 19 (KRT19) [20] were detectable in organoid lysates, but not in the liver tissue. In contrast, neither albumin nor cytochrome P450 3A4 (CYP3A4) protein expression could be observed in the organoids. Interestingly, only low expression of LCN2 could be detected in the organoids as well as in the livers. Subsequently, the stability of some markers was examined during long-term cultivation (Figure 4B). The organoids showed stable expression of *SOX9*, the stem cell marker leucine-rich repeat containing G protein-coupled receptor 5 (*LGR5*) [21], *HNF4a*, the epithelial cell adhesion molecule (*EPCAM*) [22], and especially *LCN2*, which remained stable over 6 passages. Glyceraldehyde-3-phosphate dehydrogenase (*GAPDH*) expression is shown as a reference gene.

Previous studies using various animal models have already shown that LCN2 can be induced in hepatocytes, particularly by inflammatory cytokines and LPS [1,4,23,24]. Thus, the effect of these stimulants on LCN2 protein and mRNA expression was investigated in the generated human organoids. Our data demonstrate that LCN2 expression was induced particularly by treatment with interleukin-1β (IL-1β) (Figure 4C,D). A less pronounced induction was observed in protein and mRNA levels after treatment with tumor necrosis factor α (TNF-α). Interestingly, combining the two cytokines resulted in a more pronounced LCN2 induction than treatment with IL-1β or TNF-α alone. Surprisingly, neither interleukin-6 (IL-6) nor LPS treatment altered LCN2 protein or mRNA expression. Previous results have shown that heat shock protein 90 (HSP90) and β-actin were best suited as housekeepers for organoids, as they were expressed most stably (Figure S1).

Next, NGS data from male and female organoids reflect the protein expression of *SOX9*, *HNF4α* and *CYP3A4* additionally on the transcriptional level (Table 1), where *ALB* expression was also detectable. In addition to *CYP3A4*, organoids barely expressed other CYP enzymes like *CYP2C19* and *CYP2D6*. The bile acid transporters *ABCB11* (BSEP) and *SLC10A1* (NTCP) showed no detectable transcripts per million (TPM).

**Figure 5:**
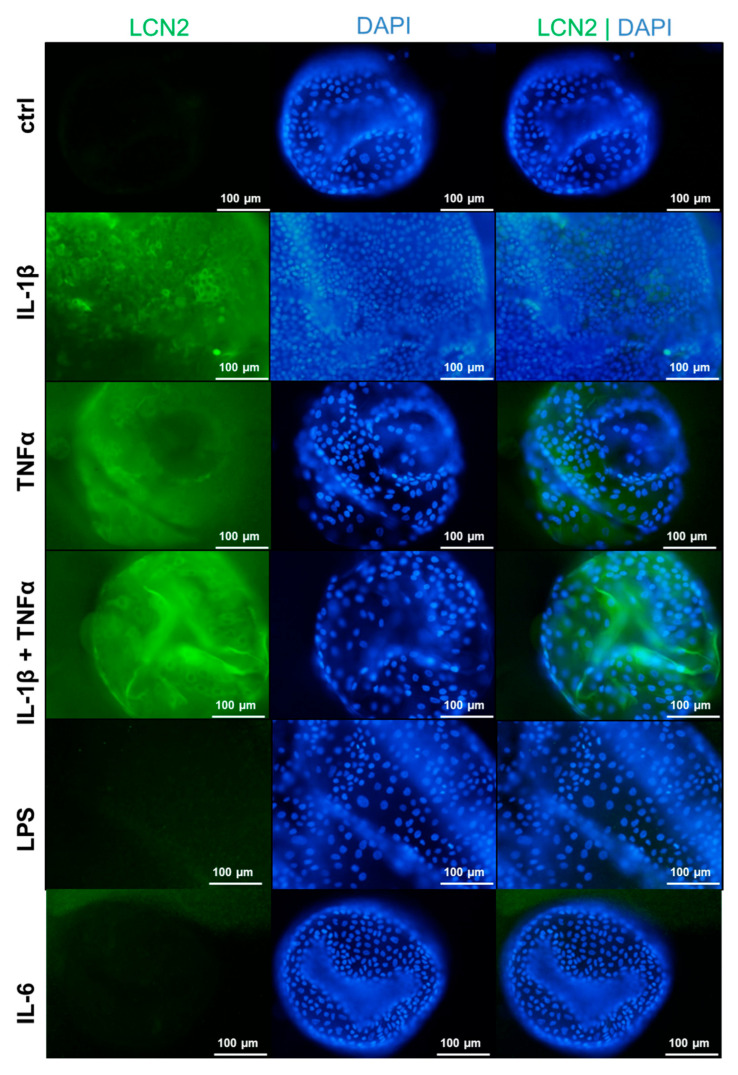
Immunofluorescence staining of LCN2 under inflammatory conditions. LCN2 expression (green) was visualized after 24 h of stimulation. Nuclei were counterstained with DAPI (blue). Organoids were stimulated with cytokines at the following concentrations: 2.5 ng/mL IL-1β, 10 ng/mL TNF-α, 10 ng/mL IL-6, and 10 µg/mL LPS. Fluorescence images were captured using a Nikon Eclipse 80i microscope at a magnification of 200×. Representative images are displayed with a scale bar indicating 100 µm.

To visualize the altered LCN2 expression in the organoids under inflammatory conditions, immunofluorescence staining was performed. The immunofluorescence staining clearly confirmed that the expression of LCN2 is induced by IL-1β, TNF-α, and a combination of both cytokines (Figure 5).

In addition, IHC was performed as additional visualization of LCN2 expression after treatment with IL-1β in the organoids (Figure S2). The specific brownish staining clearly showed the LCN2 expression in the highly epithelial organoid structures under inflammatory conditions. The LCN2 expression was mostly localized in the cytoplasm of organoids (Figure S3). However, the fact that LCN2 is a secreted protein is evident through staining of the extracellular matrix as well as secretion of LCN2 into the cell culture supernatant (Figure S4). These findings demonstrate that patient-derived organoids barely express LCN2 as seen in healthy liver tissues. In contrast, under inflammatory conditions, LCN2 was strongly upregulated.

### 3.4. Signaling Pathways Involved in the Cytokine-Induced LCN2 Expression

**Figure 6:**
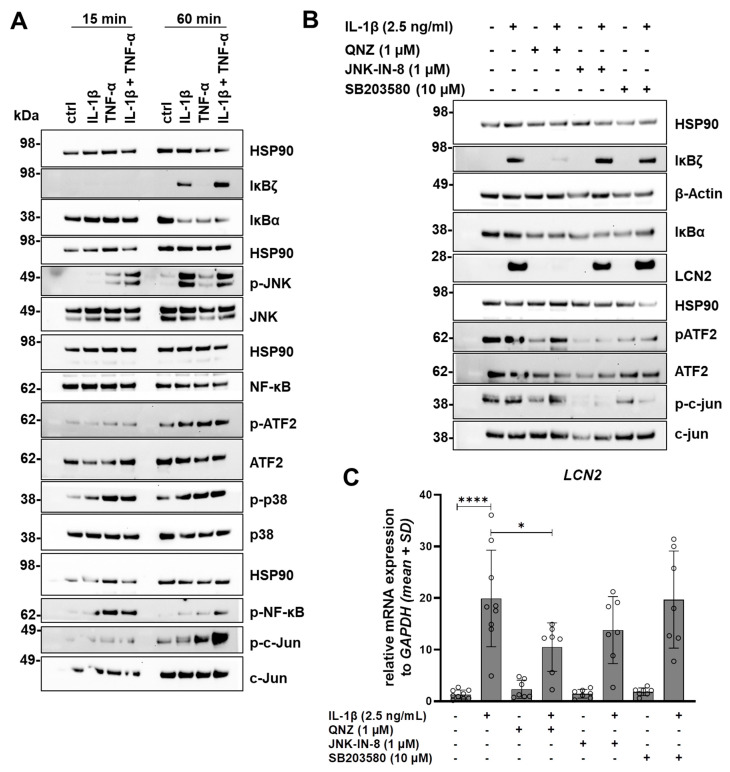
Downstream signaling pathway activation and inhibition. (**A**) Stimulation of liver organoids with LCN2 induces inflammatory cytokines for 15 min and 1 h to detect activated downstream pathways through phosphorylation or activation of selected proteins. Representative membranes are shown (n = 6). HSP90 protein expression was used as an internal control. (**B**) NF-κB inhibitor QNZ, JNK inhibitor JNK-IN-8 and p38 inhibitor SB203580 were utilized to inhibit the signaling pathways before organoids were stimulated with 2.5 ng/mL IL-1β for 24 h. Protein expression of downstream targets and LCN2 was analyzed after 24 h. Representative Western blot images are shown (n = 6). (**C**) *LCN2* mRNA expression was measured after 24 h of stimulation with IL-1β and treatment with the respective inhibitors. Data is presented as mean ± SD. Multiple comparisons of data were made to IL-1β-treated samples. Statistical significances (n = 6) are indicated with asterisks * *p* < 0.05, **** *p* < 0.0001.

Having established that human liver organoids, like primary hepatocytes and known immortalized cell lines, express LCN2 in response to stimulation [1,11], our investigation then focused on the downstream signaling pathways induced by IL-1β and TNF-α. The study centered on the three central signaling mediators: c-Jun-N-terminal kinase (JNK), mitogen-activated protein kinase p38 (p38), and nuclear factor-κB (NF-κB). To achieve this, liver organoids were stimulated with LCN2-inducing cytokines for 15 and 60 min. Activation or deactivation was then analyzed by examining the expression of proteins associated with the aforementioned signaling pathways (Figure 6A). Firstly, looking at the NF-κB signaling pathway, it is evident that all three treatments lead to the phosphorylation of NF-κB after just 15 min. However, this phosphorylation and subsequent activation decreased after 60 min. In relation to NF-κB phosphorylation, after 60 min, there was an IL-1β-dependent induction of NF-κB inhibitor zeta (IκBζ), which interacts with NF-κB in the nucleus and affects transcriptional activity [25]. This was accompanied by a decrease in the inhibitor of κB alpha (IκBα), which is ubiquitinated by pro-inflammatory cytokines. This ubiquitination allows the proteasome to degrade IκBα, resulting in the activation of NF-κB [26]. However, the decrease in IκBα was also observed with TNF-α stimulation, although there was no induction of IκBζ.

In addition to the NF-κB signaling pathway, IL-1β and TNF-α are linked to the activation of mitogen-activated protein kinases (MAPKs), particularly JNK and p38. It was evident that p38 is phosphorylated to a similar extent by all three stimuli after just 15 min, a level that was maintained for 60 min (Figure 6A). On the other hand, JNK phosphorylation was induced by TNF-α after 15 min, but was delayed and only became detectable after 60 min with IL-1β stimulation. However, combining both cytokines resulted in a stronger induction after 15 min, which remained constant up to 60 min. This activation through phosphorylation was also evident in the downstream targets c-Jun and activating transcription factor 2 (ATF2), which were observed after 60 min. This suggests that, in addition to NF-κB, MAPK signaling, specifically p38 and JNK, is also activated by the selected inflammatory cytokines and their combination.

Subsequently, it was necessary to investigate which of the induced signaling pathways might be involved in LCN2 expression (Figure 6B). NF-κB signaling was inhibited using the reversible NF-κB transcriptional activation inhibitor QNZ [27]. JNK signaling was blocked using the irreversible JNK-IN-8 inhibitor [28]. p38 signaling was inhibited using SB203580, which suppresses the downstream activation of MAPK-activated protein kinase 2 (MAPKAPK2) [29]. Established concentrations of the inhibitors according to Schröder et al. were used [30]. This was initially limited to IL-1β as a strong LCN2-inducing stimulus. Downstream protein expression, as well as LCN2 protein and mRNA expression, were analyzed 24 h after stimulation. Figure 6B shows that all three inhibitors lead to a drastic reduction in downstream signaling targets, such as IκBζ (QNZ), pATF2, and p-c-Jun (JNK-IN-8, SB203580), whereas c-Jun and ATF2 remained unaffected by the inhibitors. Interestingly, IκBα seems to be slightly reduced by all three inhibitors, even though it is an NF-κB inhibitor itself. LCN2 protein expression, on the other hand, was not affected by the p38 inhibitor, but appears to be slightly reduced by JNK inhibition, as confirmed by *LCN2* expression (Figure 6C). Although LCN2 protein expression was completely inhibited by QNZ, mRNA expression was only reduced by half. Taken together, our findings clearly demonstrated that IL-1β-induced LCN2 expression is mediated in an NF-κB-dependent manner.

## 4. Discussion

During the last decade, human liver organoids have been established as a reliable in vitro model, bridging the gap between conventional 2D cell culture and in vivo studies [31]. Such in vitro models are lacking in research on LCN2 biology, which is crucial in both physiological and pathological processes in the liver [5,6,8]. Therefore, this study aimed to establish human patient-derived liver organoids as an in vitro model for research on LCN2, including the involved signaling pathways.

During the study, we successfully isolated organoids from human liver biopsies with self-renewing potential. Organoids could be cultivated for up to 14 passages, circumventing the problem of primary hepatocytes lacking proliferative potential [13]. Moreover, TEM demonstrated that organoids mirror the ultrastructure of hepatocytes in the liver and form essential structures such as bile canaliculi, distinct from established cell lines like Huh7 [32]. The formation of bile canaliculi has already been observed in iPSC-derived organoids [33,34], and their functionality has been demonstrated in organoids established from mouse livers [35]. This highlights that organoids more closely recapitulate the structure of the liver compared to conventional 2D culture using cell lines.

Comparing the protein expression profiles of human liver tissue and patient-derived organoids provides critical insights into the suitability of these organoids as an in vitro model. As expected, the organoids expressed HNF4α, emphasizing their hepatocellular characteristics. However, albumin and CYP3A4, key metabolic enzymes of the liver, were not detectable. While albumin was detectable on the transcriptional level via NGS, other metabolic enzymes, next to *CYP3A4*, were not. In contrast, SOX9 and KRT19 were expressed, highlighting that organoids maintain a more progenitor-like phenotype and do not fully recapitulate the liver protein expression pattern. Huch et al. have already described the same for human liver organoids grown in expansion medium [36]. They additionally described the stable gene expression of *LGR5*, *SOX9*, *HNF4a* and *KRT19* compared between early and late passages [36]. Moreover, in the current study, we demonstrated the stable expression over 6 passages of the epithelial marker *EPCAM* as well as *LCN2.* Nevertheless, studies have shown that altering the culture conditions of organoids can change the expression of proteins and genes, such as albumin and CYP3A4, thereby resembling primary tissue more closely [36]. However, it has to be taken into consideration that changing the culture conditions leading to a more differentiated hepatic phenotype may lead to significant alterations not only in the expression of hepatocyte-specific genes but also influence inflammatory signaling [37]. This may result in an upregulation of LCN2, which has to be elucidated in future experiments.

When using patient-derived material, it is important to consider the potential for substantial biological variability. Therefore, a sufficient sample size is necessary for robust statistical analysis. Although the organoids used in this study were generated from tumor-free tissue and classified as healthy, they were obtained from individuals with underlying disease. Therefore, it cannot be ruled out that these tissues may harbor genetic alterations. In line with previous studies, only low LCN2 expression was detected in non-tumorigenic organoids and liver tissue extracts [8].

Under inflammatory conditions, mimicked by stimulation with selected cytokines, *LCN2* mRNA and protein levels significantly increased in human liver organoids. Specifically, IL-1β, TNF-α, and the combination of both led to elevated protein and mRNA levels compared to unstimulated control conditions. Previous studies have shown that IL-1β can induce LCN2 expression in different hepatocyte cell lines [1,11]. Through immunofluorescence staining, the LCN2 induction mediated by IL-1β, TNF-α, and the combination of both cytokines was visualized. Overall, all cells expressed LCN2, but some to a greater extent than others. It could not be excluded that these cells are exclusively hepatocytes, which is a limitation of the present study. In future approaches, markers specific for multiple hepatic cell types, like HNF4a, KRT19, EPCAM or CD68, should be additionally stained to address this limitation.

In contrast to organoids, HepG2 cells demonstrated a strong IL-6-dependent induction of LCN2 [1]. Studies in mice lacking either the IL-6 receptor or IL-6 specifically in hepatocytes showed reduced serum LCN2 concentrations compared to control animals [4]. Additionally, murine hepatocytes produce more LCN2 in the context of acute kidney injury, which is also regulated by IL-6-mediated signaling pathways [38]. Even though our NGS data revealed the expression of the IL6 receptor, the activation of STAT3 was not ruled out in this study. Therefore, canonical STAT3 signaling was not proven and may be a reason for the lack of LCN2 induction in the organoids. Overall, the IL-6-mediated LCN2 response requires further investigation through additional experiments.

Our findings demonstrate that LPS stimulation did not alter LCN2 expression in established human liver organoids. In vitro experiments using a bacterial infection mouse model showed that LCN2 plays a critical role in controlling systemic infection [39]. Additionally, injection of LPS led to increased hepatic LCN2 expression in wild-type mice and reduced hepatocyte damage, inflammation, and fibrosis in the liver compared to LCN2 knockout mice [24]. A study by Jiang and colleagues depicted increased LCN2 protein and mRNA expression in murine primary hepatocytes stimulated with LPS as a sepsis model [40]. In contrast, complementary in vitro analyses revealed that LCN2 is mainly localized in the cytoplasm of macrophages following LPS injection [41]. Moreover, conditioned medium from LPS-stimulated macrophages was sufficient to induce LCN2 expression in hepatocyte cell lines [42], indicating that macrophages are the primary cells reacting to LPS stimulation. To gain further insights into LPS-induced LCN2 expression and the role of LCN2 during infections, also in a human system, co-culture systems of organoids and macrophages could be used in a further approach [43].

Lastly, the molecular mechanisms underlying the cytokine-mediated increase in LCN2 expression were examined. Stimulation with IL-1β, as well as TNF-α, resulted in the activation of the NF-κB, JNK, and p38 signaling pathways [44,45]. Inhibition of these pathways showed that IL-1β-induced *LCN2* mRNA and protein expression is mainly mediated by NF-κB. Inhibition of NF-κB signaling reduced *LCN2* expression by half, while protein expression was completely inhibited. This may be due to post-transcriptional regulation or delayed transcriptional kinetics. Due to limited availability of material only onetime point was chosen for mRNA analysis. At other time points, the mRNA could potentially be completely inhibited as well, which should be analyzed in future studies.

The NF-κB-dependent regulation of LCN2 seems to be conserved not only across species in hepatocytes but also among diverse cell types and tissues. In mice, IL-1β activated the canonical NF-κB signaling pathway, an effect that was eliminated by treatment with QNZ, an NF-κB inhibitor [1,24]. A similar pattern was observed in the hepatocyte-derived cell line HepG2 [1]. Additionally, the upregulation of this signaling pathway in murine livers led to increased LCN2 expression [46]. NF-κB-mediated LCN2 induction has also been described in the context of myeloproliferative neoplasms, human colorectal cancer, and prostate cancer, further supporting NF-κB as a conserved central component of the LCN2 regulatory network [30,47,48]. On the other hand, although JNK signaling has been linked to LCN2 expression, inhibiting this pathway in liver organoids only resulted in a slight, non-significant reduction, suggesting that JNK is unlikely to be the primary driver of LCN2 induction [30,49].

For the first time, this study investigated LCN2 using patient-derived liver organoids. This model provides a starting point for a more detailed analysis of LCN2 function in liver pathologies. LCN2 has been proposed as a potential biomarker for NAFLD and liver pathologies, consistent with reports demonstrating its upregulation in both NAFLD and HCC [1,11,50,51]. Moreover, LCN2 is thought to exert a hepatoprotective role in the context of MASH, although most existing evidence derives from in vivo models [8]. In recent years, MASH, fatty liver disease, and steatohepatitis organoids have been established as robust in vitro systems capable of recapitulating key features, including lipid accumulation, inflammatory responses, and fibrotic signaling [52,53,54]. Parallel advances have also enabled the development of liver cancer organoids that model molecular and phenotypic characteristics of HCC [55,56,57]. Especially in HCC, organoids derived from both tumor tissue and matched tumor-free tissue from the same patient could be compared to more precisely elucidate the molecular mechanisms, such as dysregulated signaling pathways and the role of LCN2 in cancer progression. It is likely that the expression pattern differs markedly between healthy organoids and those derived from MASLD, MASH, and HCC, highlighting the need for further studies. Furthermore, organoids serve as a platform for the identification of additional drugs for the treatment of HCC. The potential for anti-LCN2 therapy has already been suggested [58]. Future studies could focus on the development of such a therapy and use organoids as a tool for the development of personalized medicine and drug testing [31]. Therefore, these organoid platforms provide physiologically relevant systems to dissect the mechanistic involvement of LCN2 in the context of liver pathologies, thereby expanding opportunities for translational research. The heterogeneity of patient-derived samples is often a major limitation in experimental design. In the present study, however, all isolates displayed comparable effects on LCN2 expression, indicating that inter-individual variability did not significantly influence the experimental outcomes. These findings underscore the utility of liver organoids as a robust model system that offers distinct advantages over primary hepatocytes and conventional cell lines. From a methodological perspective, organoids provide several practical and experimental benefits compared to primary hepatocytes. They can be cryopreserved and successfully re-established, enabling long-term culture [14]. In addition, organoids are suitable for genetic modification, including CRISPR/Cas9-based genome editing, thereby providing a replacement 3R-model to various LCN2-knockout mouse models [14,59,60]. Their use therefore represents an advantageous alternative in line with the 3R principles, particularly for investigations involving LCN2 function in the liver. Compared with established hepatocellular carcinoma–derived cell lines such as Huh7, HepG2, or Hep3B, all originating from male patients, organoids can be generated from non-malignant liver tissue and from donors of both sexes. This is of particular significance, as previous studies have demonstrated discrepancies in LCN2 expression between sexes in murine models, predominantly within the reproductive system but also in the liver [17]. These findings are especially relevant in the context of obesity, insulin resistance, and hepatic steatosis [61].

Furthermore, the study confirms previous research showing that organoids and spheroids are appropriate pre-clinical models for replicating aspects of hepatic complexity for disease modeling and toxicity assessment [62,63,64,65].

## 5. Conclusions

Overall, we were able to generate self-renewing progenitor-like hepatic patient-derived organoids that resemble the LCN2 expression of liver tissue. Organoids are a valuable tool for further research on physiological and pathophysiological processes, offering advantages compared to primary hepatocytes and cell lines. These advantages include ongoing proliferative capacity, cryopreservation and thawing of organoids, as well as tumor-free genetic background of samples. In addition, these generated organoids present a valuable tool to study LCN2 biology and associated signaling mechanisms. We found that LCN2 expression could be increased through the inflammatory cytokines IL-1β and TNFα, while IL-6 and LPS, as LCN2 inducers, require further research. On a molecular level, inflammatory cytokines led to activation of the already known signaling pathway mediators NF-κB, JNK and p38, with IL-1β-induced LCN2 expression mainly driven by NF-κB signaling pathway activation. From a clinical perspective, patient-derived organoids provide a platform for further studies on LCN2, particularly in relation to metabolic diseases such as MASLD and MASH, as well as HCC and the development of possible therapies, especially personalized medicine.

## Figures and Tables

**Table 1:** Expression of selected genes in human patient-derived organoids isolated from three male and three female patients.

Gene	Gene Description	Gene Id	TPM					
*ABCB11*	ATP-binding cassette subfamily B member 11	ENSG00000073734.10	1.55	11.60	0.89	3.92	6.40	3.58
*ACTB*	Actin beta	ENSG00000075624.17	7946.45	6300.09	9235.32	9689.05	7606.34	7433.09
*ALB*	Albumin	ENSG00000163631.19	587.39	613.39	600.69	427.23	434.00	530.94
*CD163*	CD163 molecule	ENSG00000177575.13	0.00	1.29	0.00	0.00	0.91	0.00
*CD68*	CD68 molecule	ENSG00000129226.14	26.31	94.07	41.95	69.57	105.99	66.81
*CYP2C19*	Cytochrome P450 enzyme 2C19	ENSG00000165841.11	7.74	20.62	0.00	13.72	11.88	11.93
*CYP2D6*	Cytochrome P450 enzyme 2D6	ENSG00000100197.23	3.87	15.46	16.07	13.72	8.22	8.35
*CYP3A4*	Cytochrome P450 enzyme 3A4	ENSG00000160868.16	1.55	6.44	0.00	1.96	6.40	0
*EPCAM*	Epithelial cell adhesion molecule	ENSG00000119888.11	2603.41	2892.97	2604.49	2757.38	3092.79	2768.02
*HNF4A*	Hepatocyte nuclear factor 4 alpha	ENSG00000101076.20	215.92	298.96	182.97	327.28	316.13	217.15
*IL6R*	Interleukin 6 receptor	ENSG00000160712.13	56.49	33.50	34.81	44.09	33.81	73.97
*ITGAM*	Integrin subunit alpha M (CD11b)	ENSG00000129226.14	0	0	0	0	0	1.19
*KRT19*	Keratin 19	ENSG00000171345.13	14,905.39	11,017.75	15,418.09	16,558.00	15,492.27	13,313.95
*LGR5*	Leucine rich repeat containing g protein-coupled receptor 5	ENSG00000139292.13	52.63	128.86	88.36	78.39	70.35	44.15
*SLC10A1*	Solute carrier family 10 member 1	ENSG00000100652.5	0	0	0	0	0	0
*SOX9*	SRY Box transcription factor 9	ENSG00000125398.8	2233.49	2376.23	2062.71	2009.73	1482.89	2943.41
*STAT3*	Signal transducer and activator of transcription 3	ENSG00000168610.17	465.12	413.65	431.11	422.33	419.38	462.93
*GAPDH* ^1^	Glyceraldehyde-3-phosphate dehydrogenase	ENSG00000111640.15	23,910.54	24,358.90	19,250.75	20,466.74	22,992.63	26,866.52

^1^ For the comparison of transcript levels of the listed genes, the expression of the housekeeping gene glyceraldehyde-3-phosphate dehydrogenase (*GAPDH*) is displayed. TPM stands for Transcripts Per Million.

## Data Availability

The original contributions presented in the study are included in the article and Supplementary Material; further inquiries can be directed to the corresponding authors.

## Supplementary Materials

The following supporting information can be downloaded at: https://www.mdpi.com/article/10.3390/cells15030216/s1, Figure S1: Expression of different potential housekeeping genes in hepatic organoids; Figure S2: Immunohistochemistry of LCN2 in patient-derived organoids; Figure S3: Immunofluorescence staining of human patient-derived liver organoids; Figure S4: LCN2 protein expression in the cell culture supernatant of human patient-derived liver organoids; Table S1: Antibodies used for Western blot analysis; Table S2: Primers used for RNA analysis [66,67,68,69,70]; Video S1: Live cell imaging of patient-derived liver organoids over 72 h.

## Abbreviations

The following abbreviations are used in this manuscript:

ATF2	Activating transcription factor 2
CK19	Cytokeratin 19
CYP3A4	Cytochrome P450 3A4
DAPI	4′,6-diamidine-2-phenylindole
ECM	Extracellular matrix
EPCAM	Epithelial cell adhesion molecule
GAPDH	Glyceraldehyde-3-phosphate dehydrogenase
HCC	Hepatocellular carcinoma
HNF4α	Hepatocyte nuclear factor 4 alpha
HSP90	Heat shock protein 90
IL	Interleukin
IκBα	Inhibitor of kappa B alpha
IκBζ	Nuclear factor kappa B inhibitor zeta
JNK	c-Jun-N-terminal kinase
LCN2	Lipocalin-2
LGR5	Leucine-rich repeat-containing G-protein-coupled receptor 5
LPS	Lipopolysaccharide
MAPK	Mitogen-activated protein kinases
MAPKAPK2	Mitogen-activated protein kinase-activated protein kinase 2
MASH	Metabolic dysfunction-associated steatohepatitis
MASLD	Metabolic dysfunction-associated steatotic liver disease
NF-κB	Nuclear factor kappa B
NGS	Next-generation sequencing
NGAL	Neutrophil gelatinase-associated lipocalin
p38	Mitogen-activated protein kinase p38
RT-PCR	Reverse transcriptase PCR
RT-qPCR	Real-time quantitative PCR
SOX9	SRY-box transcription factor 9
TEM	Transmission electron microscopy
TNF-α	Tumor necrosis factor alpha
