# Supplementary material for: Human Liver Organoids as an Experimental Tool to Investigate Lipocalin-2 in Hepatic Inflammation
Source: Cells. 2026 Jan 23;15(3):216. doi: 10.3390/cells15030216 (PMC12896775; doi:10.3390/cells15030216)
Supplement: Supplementary file 1 [file cells-15-00216-s001.zip › Tables S1-S2 and Figures S1-S4.pdf]

Supplemental Material

Human Liver Organoids as an Experimental Tool to Investigate Lipocalin-2 in Hepatic Inflammation

Katharina S. Hardt, Robert F. Pohlberger, Diandra T. Keller, Eva M. Buhl, Florian W.R. Vondran, Anjali A. Roeth, Ralf Weiskirchen and Sarah K. Schröder-Lange

Table S1. Antibodies used for Western blot analysis

**Primary antibodies**	**Cat.-No**	**Company**	**Dilution**	**Clonality**
Albumin	#4929	Cell Signaling Technology	1000	r pAb
ATF2	#9226	Cell Signaling Technology	1000	r pAb
c-Jun	#9165	Cell Signaling Technology	1000	r mAb
Cyclophilin A	#2175	Cell Signaling Technology	1000	r pAb
CYP3A4	18227-1-AP	Proteintech	1000	r pAb
Cytokeratin 19 (KRT19)	SAB5600252	Sigma Aldrich	1000	r mAb
HNF4α	sc-6556	Santa Cruz	1000	g pAb
HSP90	#4877	Cell Signaling Technology	1000	r mAb
IκBα	sc-371	Santa Cruz	1000	r pAb
IκBζ	#9244	Cell Signaling Technology	1000	r pAb
Lipocalin 2 (LCN2)	AF1757	R&D Systems	1000	g pAb
NF-κB (p65)	sc-8008	Santa Cruz	1000	m mAb
p38	612168	BD Pharmingen	1000	m pAb
p-ATF2 (Thr71)	#9221	Cell Signaling Technology	1000	r pAb
p-c-Jun (Ser73)	#3270	Cell Signaling Technology	1000	r mAb
p-NF-κB (Ser536)	#3033	Cell Signaling Technology	1000	r mAb
p-p38 (T180/Y182)	612288	BD Pharmingen	1000	m mAb
P-SAPK/JNK (Thr183/Thr185)	#4668	Cell Signaling Technology	1000	r mAb
SAPK/JNK	#9252	Cell Signaling Technology	1000	r pAb
SOX9	#82630	Cell Signaling Technology	1000	r mAb
β-Actin	A5441	Sigma-Aldrich	10000	m mAb
**Secondary antibodies**	**Cat.-No**	**Company**	**Dilution**	**Clonality**
goat anti-rabbit IgG (H+L), HRP	#31460	Invitrogen	5000	g
goat anti-mouse IgG (H+L), HRP	#31430	Invitrogen	5000	r
mouse anti-goat IgG (H+L), HRP	#31400	Invitrogen	5000	m

Abbreviations: g, goat; m, mouse; mAb, monoclonal antibody; pAb, polyclonal antibody; r, rabbit.

Table S2. Primers used for RNA analysis

Gene	Accession No	Forward primer (5′->3′)	Location	Reverse Primer (5′->3′)	Location	Amplicon Size	Reference[1]
SOX9	NM_000346.4	GCTCTGGAGACTTCTGAACGA	795-815	CCGTTCTTCACCGACTTCC	907-926	132	[66]
LGR5	NM_001277226.2	CGGGAAACGCTCTGACATACAT	576-597	TGAAACAGCTTGGGGGCACATA	737-758	183	[67]
HNF4a	NM_000457.6	GAGCTGCAGATCGATGACAA	966-985	TACTGGCGGTCGTTGATGTA	1098-1117	152	[68]
EPCAM	NM_002354.3	GCTGGCCGTAAACTGCTTTG	294-313	TAAAGAGCCCGCTCTCATCG	492-511	218	[69]
LCN2	NM_005564.5	CAGCATGCTATGGTGTTCTTCA	482-503	CGTCGATACACTGGTCGATTG	665-684	184	NCBI Primer Design[2]
GAPDH (RT-PCR)	NM_001289726.2	ACTGCCACCAAGACTG	650-668	CACCACCCTGTTGCTGTAG	1063-1081	432	[70]
GAPDH (RT-qPCR)	NM_001289746.2	AGCCACATCGCTCAGACAC	57-75	GCCCAATACGACCAAATCC	104-122	66	[30]

[1] For references, please see main document; [2] The NCBI Primer Design tool is available online at: https://www.ncbi.nlm.nih.gov/tools/primer-blast/

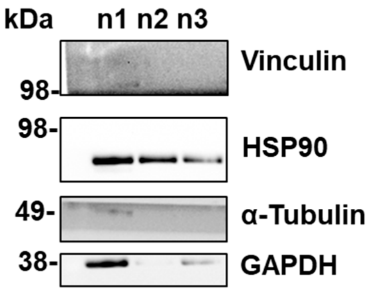

Figure S1. Expression of different potential housekeeping genes. n1, n2, and n3 represent protein extracts obtained from different hepatic organoid cultures.

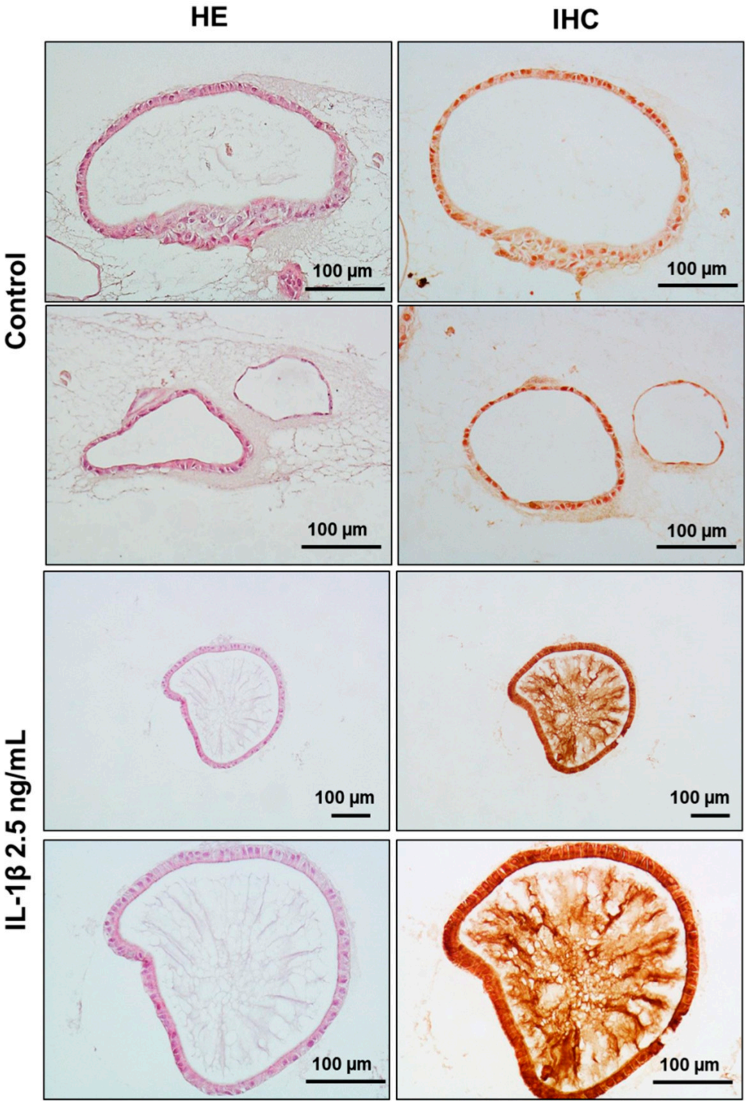

Figure S2. Immunohistochemistry of LCN2 in patient-derived organoids. Hematoxylin and eosin (HE) staining (left panels) and immunohistochemistry (IHC) staining of LCN2 (right panels) was performed in serial sections of patient-derived human liver organoids, both with and without the addition of IL-1β as a stimulus. The scale bar equals 100 µm.

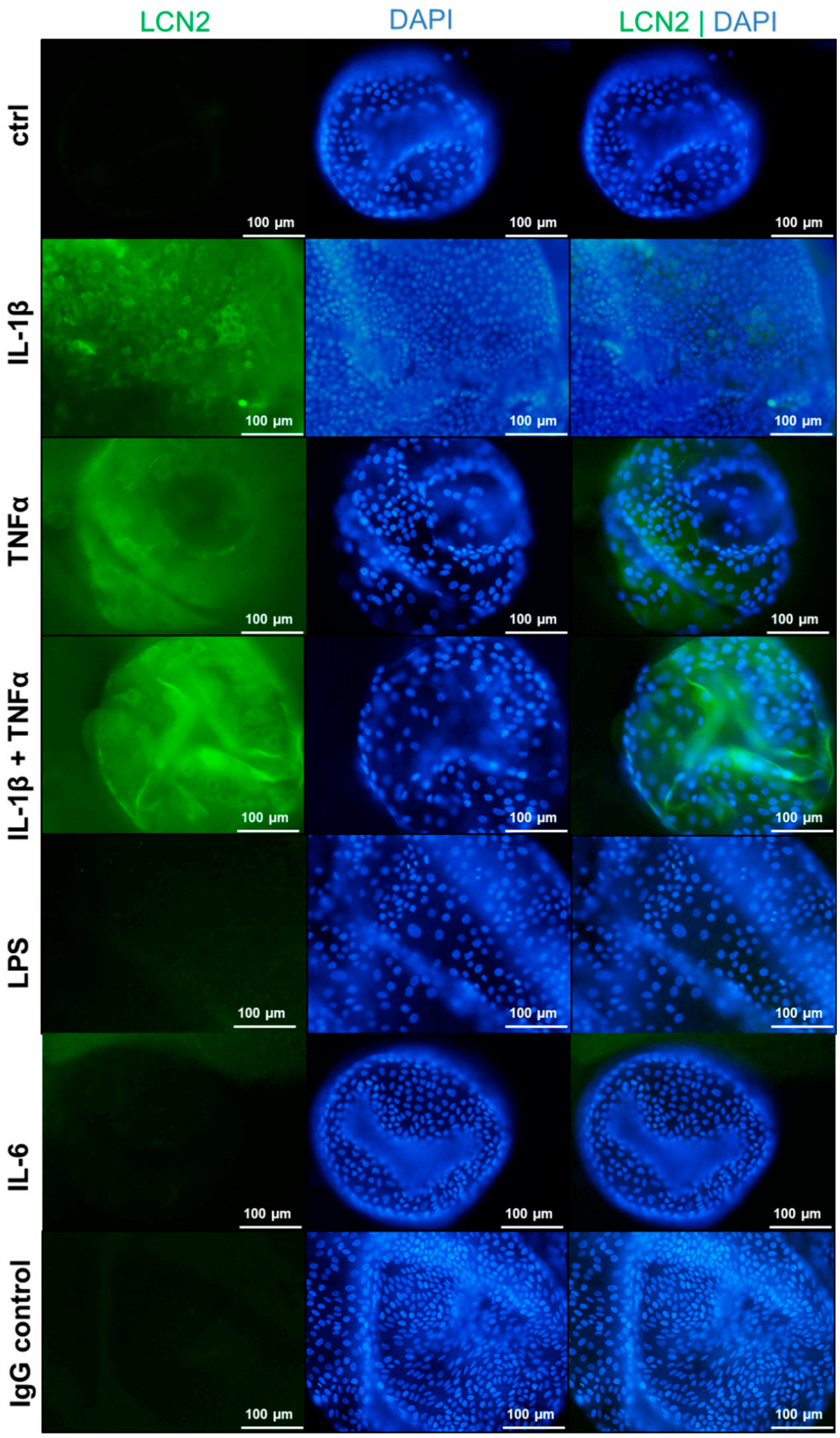

Figure S3. Immunofluorescence staining of human patient-derived liver organoids. LCN2 expression is shown in green, with nuclei counterstained with DAPI (blue). The scale bar equals 100 µm. Pictures were taken at 200x magnification.

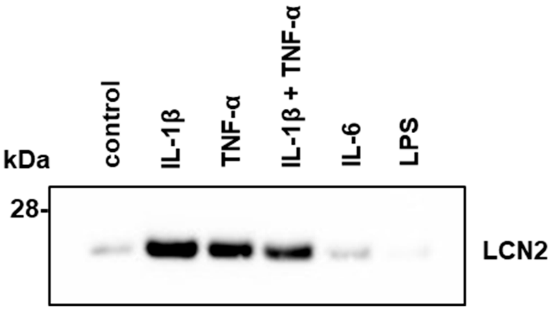

Figure S4. LCN2 protein expression in the cell culture supernatant of human patient-derived liver organoids.
